# When somatic attention creates sensation: a new class of bodily experience

**DOI:** 10.3389/fnhum.2026.1884618

**Published:** 2026-08-18

**Authors:** Daria Iwasinski, Olivia Delahaye, Florian Beissner

**Affiliations:** 1 Insula Institute for Integrative Therapy Research, Hannover, Germany; 2 Hannover Medical School, Institute for Neuroradiology, Hannover, Germany; 3 University of Witten/Herdecke, Witten, Germany

**Keywords:** somatic attention, referred sensation, predictive coding, top-down processing, restricted environmental stimulation, floatation tank, body awareness, sensation drawing

## Abstract

Attention is typically regarded as a modulatory process that amplifies or suppresses existing sensory input. Here we tested whether inward-directed (somatic) attention can instead generate bodily sensations. In a floatation-based sensory deprivation paradigm (restricted environmental stimulation), 100 healthy adults were assigned to three groups: focused somatic attention with a light tactile anchor on a finger or toe (FSA, *n* = 50), natural somatic attention (NSA, *n* = 25), or passive somatic awareness (PSA, *n* = 25). Participants recorded the quality, intensity, and location of any sensations as digital body drawings, which we analyzed quantitatively and with descriptor clustering and contour-based shape analysis. Focused somatic attention produced more frequent, more complex, and more spatially structured sensations than the natural control conditions, though of lower intensity and smaller extent. Most FSA sensations (77%) were referred to sites remote from the anchor, were predominantly ipsilateral, and fell into distinct descriptor families and two geometric forms—compact patches and elongated, pathway-like trajectories spanning multiple joints. These findings demonstrate that somatic attention is not merely modulatory but generative: directed toward minimal, localized tactile input, it can construct coherent referred bodily experiences at unstimulated sites and organize their spatial structure—consistent with a predictive-coding account in which weak bottom-up input tilts the balance toward top-down prediction.

## 1 Introduction

Attention is a fundamental capacity of the mind, shaping how organisms select, weight, and interpret the continuous stream of available information. In cognitive neuroscience and psychology it is usually described as a modulatory mechanism: a filter that enhances or suppresses existing inputs and thereby optimizes behaviour (Posner and Petersen, 1990; Desimone and Duncan, 1995). In the last decades research has mostly been focusing on attention to external signals such as vision, audition, or touch. Here, attention as a selective process has been shown to sharpen thresholds, amplify signals, and alter perceptual appearance itself (Ungerleider and Kastner, 2000; Carrasco, 2011).

Recent predictive-processing accounts recast this modulatory picture. On these accounts, perception is not a passive registration of input but a process of inference, in which the brain continually predicts the causes of its sensory signals and updates those predictions against the incoming evidence, weighting each source by its estimated reliability, or precision (Friston, 2005; Clark, 2013). Within this framework, attention is understood not as a filter but as the mechanism that sets this precision, raising the gain on the prediction errors arising from the attended source (Feldman and Friston, 2010). Applied to the body, these representations are organized somatotopically (Kaas, 1997), and the somatosensory system is itself actively predictive: the brain anticipates the tactile consequences of events in a body-part-specific manner (Kilteni and Ehrsson, 2020), and such predictions can shape cortical responses even in the absence of afferent input, as evidenced by expectation-driven responses to omitted touch (Andersen and Lundqvist, 2019) and by illusory, stimulus-absent touch engaging the corresponding region of primary somatosensory cortex (Blankenburg et al., 2006). Two implications follow. Attention can bias what is perceived, and, when the incoming signal is weak or ambiguous, it increasingly governs how that signal is interpreted, allowing prior expectations to determine the quality attributed to it (Summerfield and Egner, 2009; De Lange et al., 2018). On this view, attention is potentially constitutive of perceptual content, and not merely selective among pre-existing inputs.

By contrast, much less is known about what happens when attention is directed inward. Interoception studies have shown that attention to inner signals such as heartbeat or breathing can modulate awareness, detection accuracy, and emotion (Critchley et al., 2004; Mehling et al., 2011; Garfinkel and Critchley, 2013). Research on proprioception and somatosensory monitoring demonstrates that inward attention can influence sensitivity to limb position, tactile perception, or pain (Longo et al., 2008; Tsakiris, 2017). Such approaches, however, typically assume that sensations pre-exist and that attention merely adjusts their salience.

Spontaneous or poorly localized bodily sensations, including referred sensations and paresthesias, are common features of many pain conditions, yet their perceptual mechanisms remain poorly understood.

Here, we introduce the construct of focused somatic attention, which we define as the deliberate directing of attention inward to the body and toward specific body regions. Unlike interoception, which denotes perception of specific physiological signals or conditions, the broader concept of somatic attention refers to an intentional inward focus on consciously accessible bodily experience. Therefore, it allows us to test whether attention merely modulates existing bodily signals or whether it can also generate sensations itself.

To minimize external sensory input and to provide a controlled environment for studying internally experienced phenomena (Lilly, 1977; Feinstein et al., 2018), we investigated this question under restricted environmental stimulation (REST) in a floatation tank. Participants were assigned to three groups with different attentional modulation: Focused Somatic Attention (FSA), Natural Somatic Attention (NSA), and Passive Somatic Awareness (PSA). When NSA and PSA were pooled, they are referred to collectively as the Natural Control (NC) condition. A glossary of key terms used throughout this article is provided in the Supplementary material.

We show that focused somatic attention can generate structured, pathway-like bodily sensations extending beyond the attended body region. These findings suggest that attention is not solely modulatory but can actively shape the emergence and spatial organization of somatosensory experience, a mechanism that may contribute to the generation of spontaneous or referred bodily sensations.

## 2 Methods

### 2.1 Study design

**Figure 1:**
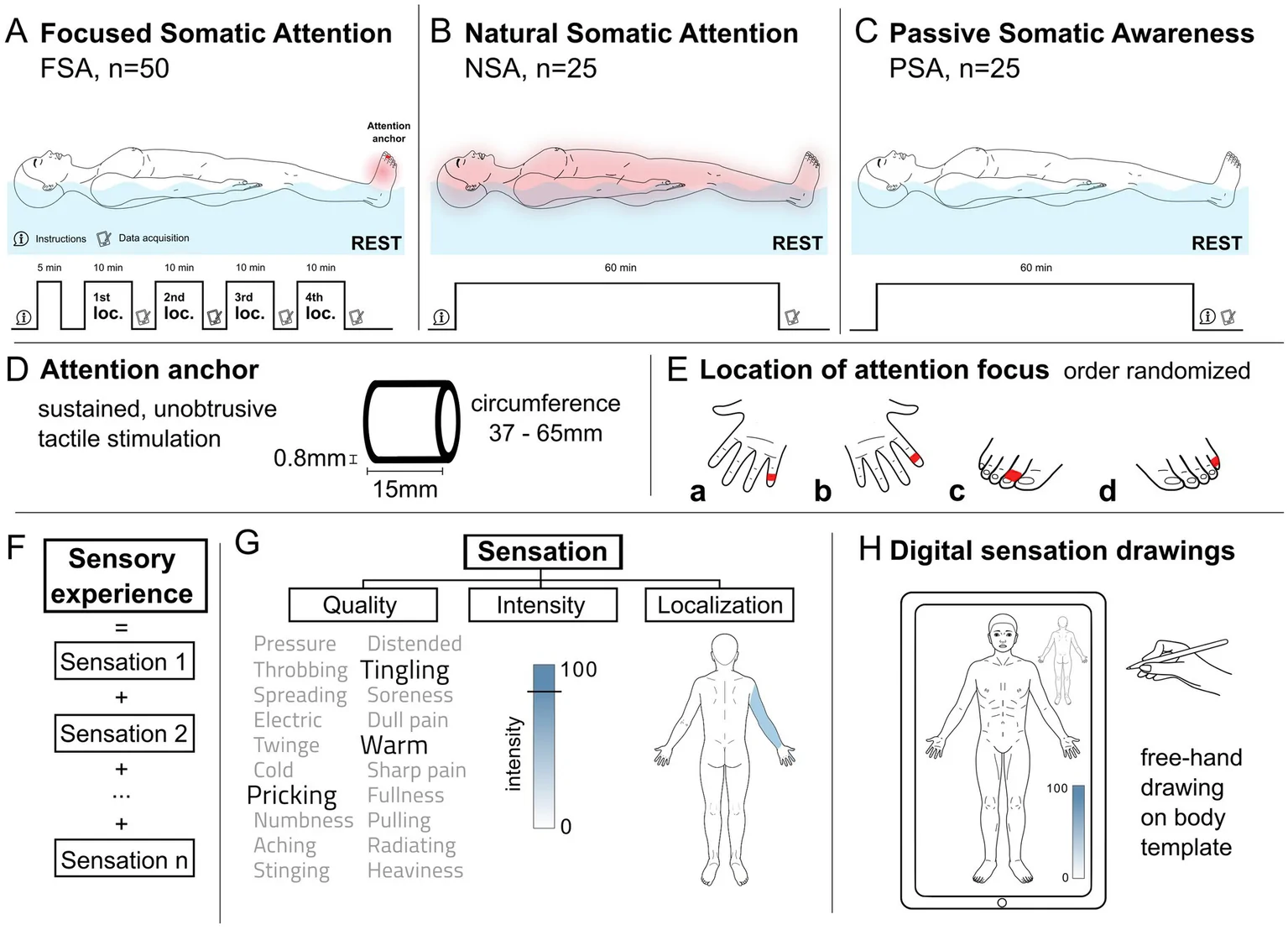
Experimental design. We compared three groups differing in the level of attention guidance: **(A)** The Focused Somatic Attention (FSA) group received verbal instructions and attention anchors at four peripheral locations; **(B)** The Natural Somatic Attention (NSA) group received the same verbal instructions but without attention anchors; **(C)** The Passive Somatic Awareness (PSA) group received neither instructions nor attention anchors. All sessions took place in a floating tank under Restricted Environmental Stimulation (REST) conditions. **(D)** Locations of attention focus: (a) right little finger, (b) left index finger, (c) right index toe, and (d) left little toe. **(E)** Attention anchors consisted of individually fitted rubber rings, which FSA participants wore successively at one of four locations for the duration of 10 min. **(F)** Physical sensations reported within a single condition were collectively treated as one sensory experience. **(G)** Participants reported their individual sensations along three dimensions: Quality (verbal descriptors); intensity (visual analogue scale); and localization (marked on a body template). **(H)** Sensory experiences were documented in a single workflow as annotated freehand digital sensation drawings on a body template.

To investigate how sensory experiences vary with different levels of attention and awareness, we implemented a three-group design differing in type and degree of attentional modulation (Figures 1A–C). All groups were studied in a floating tank under Restricted Environmental Stimulation (REST) (Lilly, 1977; Feinstein et al., 2018), minimizing somatosensory input and external distractions.

The Focused Somatic Attention (FSA) group received inward-focusing instructions combined with a tactile attention anchor, a physical cue directing attention to specific peripheral sites (fingers and toes). The Natural Somatic Attention (NSA) group received the same inward-focusing instructions but without an anchor. The Passive Somatic Awareness (PSA) group received neither instructions nor anchors and were unaware of participation until after their REST session. Only afterward were they asked whether they had noticed any sensations during floating, and those who responded affirmatively reported their experiences.

For certain analyses, data from NSA and PSA were pooled; these groups are referred to collectively as the Natural Control (NC) condition.

### 2.2 Attentional modulation

Participants in the FSA group received verbal instructions to *“float for 10 minutes with the ring, tune into yourself and notice what physical sensations you feel, if any, without interpreting them”* (for details see Supplementary material). During each period an attention anchor (“the ring”) was applied to one of four peripheral body regions in a pseudo-randomized order: (a) the right little finger, (b) the left index finger, (c) the right second toe (index toe), and (d) the left little toe (see Figure 1D).

Attention anchors (see Figure 1E) were 15 millimeter wide rings made from butyl rubber, crafted from 0.8-millimeter-thick bicycle inner tubes (“Schwalbe No. 17,” Ralf Bohle GmbH, Reichshof, Germany). Ring circumferences (37–65 mm, 1-mm steps) were customized per participant. The selection was guided by the instruction: “The ring should fit tightly without slipping or cutting in. You should be able to feel it clearly without discomfort.” The latter was important as we wanted to avoid participants experiencing pain or any kind of discomfort.

Participants in the NSA group received similar inward-focusing instructions to *“occasionally tune into yourself and notice any sensations you may have”* but were not given any tactile anchors to guide their attention. Finally, the PSA group was studied retrospectively under natural conditions, i.e., without pre-session instructions or attention anchors.

### 2.3 Sensory deprivation

All measurements were conducted in a floating tank (Lilly, 1977) under restricted environmental stimulation (REST) conditions (Feinstein et al., 2018). The cubic floating pool, located at a local floating center (float Hannover, Hannover, Germany), measured approximately 2.4 m x 1.9 m with a water depth of 27 cm. The water in the tank was saturated with 500 kg of Epsom salt (magnesium sulfate, MgSO4), creating a buoyancy of approximately 1.2 g/L, allowing participants to float effortlessly.

The water temperature was maintained at a constant 35 °C, which matches skin temperature to prevent participants from experiencing thermal sensations. The tank was soundproof, and the only light source was a dim blue light, which remained on throughout the experiment. This setup minimized both auditory and visual sensations, ensuring a highly controlled environment for the study.

### 2.4 Participants

100 healthy adults (age ≥ 18 years in Germany) were recruited for our study. Group sizes were unequal by design. FSA, the experimental group of primary interest, was largest (*n* = 50) and, with four anchor periods per participant, contributed most observations. NSA (*n* = 25) and PSA (*n* = 25) were sized to match FSA at the participant level when pooled into the Natural Control condition. As this study is exploratory, sizes were chosen to provide a well-powered experimental group with balanced controls rather than from an *a priori* power calculation.

All participants gave written informed consent following the ethics committee of the Hannover Medical School, who had approved the study. They were compensated (50€). All participants were healthy on the day of measurement (self-report). One participant of the FSA group had incomplete data due to developing nausea after the third 10-min session.

The FSA group had additionally been screened for pre-existing conditions beforehand and undergone minimal quantitative sensory testing (see below) to exclude potential unrecognized sensory disorders. Exclusion criteria were pregnancy, breastfeeding period, epilepsy, alcohol or drug abuse, open wounds, contagious diseases, gastroesophageal reflux disease, severe motion sickness, psychiatric illnesses, incontinence, and acute skin diseases, as participants were required to float as part of the study.

Participants of the FSA group were recruited via mailing lists and public displays, while those of the NSA and PSA groups were recruited from regular customers of the float center, which they were visiting for a private float session. The assignment of participants to the NSA and PSA groups was determined by random selection regarding the timing of their approach. At their arrival to the float center, potential participants were randomly chosen to be approached either before or after their float session. Participants who were approached before the float and agreed to take part were assigned to the NSA group, while those approached after the float and who consented were assigned to the PSA group.

Sex was recorded as self-reported male or female. Recruitment aimed for approximate balance across groups (FSA: 29 female, 21 male; NSA: 14 female, 11 male; PSA: 15 female, 10 male). No further sex or gender identity data were collected. The study was not designed or powered to detect sex differences, and none were hypothesized. The mean age (± SD) was 25.6 ± 5.0 years for participants in the FSA group, 34.0 ± 6.9 years for participants in the NSA group, and 35.2 ± 10.6 years for participants in the PSA group. There was no significant difference in age between the NC groups (*p* = 0.64), however participants of the FSA group were younger than the pooled NC group (*p* < 0.001).

Participants in the FSA group were asked to abstain from alcohol for 24 h and from caffeine for 6 h prior to the experiment. In the NC group, consumption of consciousness-altering medications, alcohol, or other drugs was prohibited per float-center regulations. All participants were also instructed to avoid shaving immediately before floating, as the saline solution can cause skin irritation and discomfort.

### 2.5 Minimal quantitative sensory testing

To rule out undiagnosed sensory deficits, participants of the FSA group underwent a minimal quantitative sensory test using von Frey filaments (“OptiHair2,” Marstock/MRC Systems GmbH, Heidelberg, Germany). Participants were touched with filaments at various locations on the back of their fingers while their view of the stimulation site was blocked. The stimulation force was gradually reduced (16 mN, 8 mN, 4 mN, 2 mN, 1 mN, 0.5 mN, 0.25 mN), with each filament applied five times. Sensory detection was considered normal if all five touches with the 1 mN filament were correctly reported (Johansson et al., 1980). This criterion was met by all fifty FSA participants.

### 2.6 Data acquisition

Each participant in the FSA group underwent 4 × 10-min trials in one session; NSA/PSA contributed one natural float session each. To capture participants’ sensory experiences in detail (see Figure 1F), we asked them on every trial to report zero or more individual sensations, each as an ordered triple of quality, intensity, and location (see Figure 1G). Participants were free to indicate that no sensation occurred, or to record as many sensations as they wished, using overlapping descriptors, intensity levels, or drawing areas without restriction. This participant-driven approach yielded an unconstrained, high-resolution mapping of their sensory experience.

All three outcome streams—quality, intensity, and location—were recorded in a single workflow (see Figure 1H) within the *SymptomMapper* app (Neubert et al., 2018). Participants used a Samsung Galaxy Note 10.1 (2014 edition; SM-P600; 25.7 cm diagonal, 1,600 × 2,560 px) and the inductive S-Pen stylus (Samsung, Suwon, South Korea). All participants received standardized verbal instructions on how to record their sensations before their first drawings (see Supplementary material). Participants of the FSA group further recorded three mock sensations on the tablet as a proficiency check (see Supplementary material); all passed. No data loss occurred.

#### 2.6.1 Sensation quality (verbal descriptors)

Participants first characterized each sensation by choosing one or more descriptors from a scrollable list of 20 German descriptors (see Supplementary material). This set was assembled specifically for the present study, because no validated, general-purpose instrument exists for capturing the full range of non-nociceptive bodily sensations: established tools such as the German version of the McGill Pain Questionnaire (Stein and Mendl, 1988) are confined to pain, whereas the experiences of interest here extend well beyond it.

We provided a predefined list rather than relying solely on free-text responses to improve comparability across participants, increase the robustness of the data, and facilitate quantitative analysis; to limit any bias introduced by predefined options, participants could additionally describe sensations in their own words. Because our paradigm did not primarily involve nociceptive stimulation, the descriptors were drawn from a meta-analysis that systematically compiled sensory qualities reported across paradigms beyond pain (Beissner, 2020). From this source we retained all descriptors reported more than 100 times, which yielded the final set of 20 terms, a manageable number of response options that still ensures broad coverage of the relevant sensory qualities.

English terms were translated into German by forward–back translation, expert review, and comparison with the validated German McGill Pain Questionnaire (Stein and Mendl, 1988), yielding: tingling, numbness, warm, dull pain, heaviness, soreness, sharp pain, pricking, fullness, throbbing, aching, spreading, pressure, electric, distended, stinging, twinge, pulling, radiating, and cold (German list in Supplementary material). An “Other term” free-text option was used in only 3.8% of 551 sensations, confirming adequate coverage. The terms included: “twitching” (11 times by three subjects), “tense” (three times by three subjects), and the following terms, each of which was only reported once: “stiffness,” “dizziness,” “tightness,” “discomfort,” “slight burning,” and “like a jackhammer.” As they carried little information, they were excluded from all subsequent analyses regarding descriptors.

Selections were stored as a JSON array together with a time-stamp. No drawing was possible until at least one descriptor had been selected.

#### 2.6.2 Sensation intensity (VAS)

Immediately after descriptor selection, participants set an initial intensity on a 0–100 visual-analogue slider beneath the drawing canvas (0 = “no sensation,” 100 = “strongest imaginable”). Participants could adjust the slider as often as they wished. Each new value applied only to strokes drawn after the adjustment.

*SymptomMapper* writes the current slider value into the pixel saturation of newly drawn strokes. Thus, the final drawing contains multiple saturation levels if the participant changed the slider while sketching.

#### 2.6.3 Sensation localization (digital drawings)

Spatial data were collected as digital sensations drawings (Shaballout et al., 2019) on a sex-specific, front-and-back body outline (Neubert and Beissner, 2019). Participants sketched freely on the tablet PC with the app preventing drawing outside the template’s boundaries. Each stroke was rendered with the current intensity encoded as pixel saturation in HSV (hue, saturation, value) color space. Completed sketches were saved in as 24-bit PNGs with a resolution of 1,555 × 2,200 pixels.

### 2.7 Data analysis

All computations and data manipulations were performed using custom scripts written in Python 3.10.12, utilizing NumPy, pandas, SciPy, Pillow, OpenCV, Matplotlib, Seaborn, Statsmodels, scikit-learn and scikit-image. Custom analysis scripts are available at https://doi.org/10.5281/zenodo.17054829. Statistical analyses were conducted in jamovi 2.6.44 (The jamovi project, 2025) and complemented by custom Python scripts.

We explicitly distinguish between pre-specified analyses (group-level comparisons) and exploratory procedures (e.g., generalized sensations, shape analysis), which were developed in response to unexpected patterns in the data.

Statistical analysis included descriptive statistics, two-tailed two-sample *t*-tests, Mann–Whitney *U*-tests, *χ*^2^ tests (with Pearson residuals examined for cell-by-cell contributions), and Kruskal–Wallis tests with the Dwass–Steel–Critchlow–Fligner procedure as appropriate and Quade’s rank-based ANCOVA for age-adjusted group comparisons (Supplementary Table S1). Prior to each test, key assumptions were checked—for example, normality with the Shapiro–Wilk test and homogeneity of variances with Levene’s test, and for *χ*^2^ tests ensuring sufficient expected cell counts. Groups were pooled if no significant differences in relevant outcomes were identified between them. All statistical tests were two-tailed, with a *p* value ≤ 0.05 considered significant. When multiple comparisons were performed, *p* values were adjusted using the adaptive Storey and Tibshirani (2003) *q*-value procedure, estimating *π*₀ from the data and declaring results significant at an FDR threshold of *q* ≤ 0.05. Effect sizes were reported as Cohen’s *d* for *t*-tests and Mann–Whitney tests (using rank-biserial correlation where appropriate), odds ratios for Fisher’s exact tests, and Cramer’s *V* for *χ*^2^ tests. All descriptive statistics are reported as mean ± SD unless otherwise specified; for non-normally distributed variables, median [IQR] is reported.

Sensitivity varied with the unit of analysis. Because most between-group comparisons were performed at the level of individual sensations or shapes (section 2.7.1), these Mann–Whitney tests were, at 80% power (*α* = 0.05, two-sided), sensitive to small effects (rank-biserial *r* ≈ 0.15–0.20), and the corresponding categorical tests to Cramér’s *V* ≈ 0.12. Participant-level comparisons (*n* ≈ 50 per group) and within-participant correlations (*n* = 50) were sensitive to larger effects (rank-biserial *r* ≈ 0.33 and |*r*| ≈ 0.39, respectively).

#### 2.7.1 Group-level comparisons

To compare our experimental groups at the overall level, we analyzed differences in sensation frequency, complexity, descriptor usage, sensation intensity, and spatial distribution. Each trial from each participant was treated as an independent observation unless otherwise specified. NSA and PSA groups were first compared directly; as no significant differences were found for key outcomes, they were pooled into a single natural control (NC) group for subsequent analyses.

##### 2.7.1.1 Responder rate

For each group, participants were classified as responders if they reported at least one sensation and non-responders otherwise. Group differences in responder rate were tested with *χ*^2^ tests.

##### 2.7.1.2 Generalized sensations

During preliminary inspection of the extent data, we observed a small set of sensations that encompassed nearly the entire body template. This pattern was not anticipated in our study design and was therefore defined post-hoc as generalized sensations, operationalized as sensations covering more than two-thirds of the template. These generalized sensations were excluded from further analyses of extent and lateralization, although their occurrence was quantified and compared between groups using *χ*^2^ tests.

##### 2.7.1.3 Sensation complexity

Complexity was quantified as (i) the number of distinct sensations per sensory experience, (ii) the number of descriptors per sensation, and (iii) the number of unique descriptors per participant. For the FSA group, the latter was pooled across anchor locations. Group differences were tested using Mann–Whitney *U*-tests.

##### 2.7.1.4 Sensation intensity

Intensity values were extracted from the digital sensation drawings using the inverse of the saturation-channel encoding applied during data entry. For each sensation, the mean intensity was calculated across all non-zero pixels. Group comparisons were conducted using Mann–Whitney *U*-tests.

##### 2.7.1.5 Sensation extent and lateralization

Spatial extent was calculated as the percentage of the body template covered by colored pixels (extent metric). Laterality was quantified with a lateralization coefficient (LC) computed as (N_right − N_left) / (N_right + N_left), where N_right and N_left are the numbers of pixels on each body side. The absolute value |LC| was used to assess the degree of lateralization regardless of side.

##### 2.7.1.6 Descriptor frequencies

Selection frequencies of the 20 predefined descriptors were calculated at the sensation level. Group differences in individual descriptor usage were assessed with FDR-corrected *χ*^2^ and Fisher’s exact tests. To provide a rapid visual summary, descriptor frequencies were presented as word clouds in which font size was scaled proportionally to frequency.

##### 2.7.1.7 Spatial distributions

Spatial distributions were visualized per group using overlap maps, generated by pixel-wise summing binarized (intensity > 0) sensation masks across participants and anchor conditions. Maps were thresholded to include only regions with a minimum overlap of two masks, then normalized by the total number of masks. Color coding was applied, and group differences were assessed visually.

To assess the robustness of the principal group comparisons to the age difference between groups, age-adjusted comparisons were performed using Quade’s rank-based ANCOVA; within-group associations between age and key outcomes were assessed with Spearman correlations (Supplementary Table S1).

#### 2.7.2 Local vs. referred sensations (FSA group)

Having established that the sensations of interest occurred only in the FSA group (see previous section), all subsequent spatial analyses were restricted to this group.

Sensations were classified as local or referred based on their spatial relationship to the anchor site.

##### 2.7.2.1 Classification criteria

Sensations confined to the anchor region were labeled local. Because the anchor itself was always located on the stimulated side, local sensations were by definition ipsilateral and therefore excluded from laterality analyses. Sensations extending ≥ 20 pixels beyond the metacarpophalangeal (fingers) or metatarsophalangeal (toes) joint line were classified as referred. This threshold ensured that only sensations clearly displaced from the anchor site were considered referred.

##### 2.7.2.2 Spatial distribution analysis

For each category, group-level overlap maps were generated by summing binarized (intensity > 0) sensation masks across participants and dividing by the total number of masks in that category, as described in the above section. In addition to qualitative inspection of these distributions, we examined the characteristic pathways of referred sensations across the body.

##### 2.7.2.3 Descriptor profiles

For local and referred sensations separately, descriptor frequencies were calculated at the level of individual sensations. *χ*^2^ and Fisher’s exact tests were used to identify descriptors overrepresented in one category.

##### 2.7.2.4 Sensation intensity

Mean pixel intensity (non-zero pixels only) was calculated for each sensation and compared between local and referred categories using Mann–Whitney *U*-tests.

##### 2.7.2.5 Side dominance

Each referred sensation was classified as ipsilateral, contralateral, or bilateral relative to the anchor site, and the percentage of sensations in each category was calculated. Local sensations were, by definition, ipsilateral and therefore excluded from this analysis. A *χ*^2^ test of proportions was used to test if the distribution of side dominance categories differed significantly from chance.

#### 2.7.3 Descriptor co-occurrence and clustering

To examine the internal structure of descriptor usage in the FSA group, we analyzed descriptor co-occurrence patterns and grouped descriptors into clusters using hierarchical clustering. After assigning sensations in the FSA group to descriptor clusters, we examined whether clusters differed in their spatial, intensity, and referral characteristics.

##### 2.7.3.1 Co-occurrence matrix

A binary descriptor-selection matrix was constructed at the sensation level (rows = sensations, columns = descriptors; 1 = selected, 0 = not selected). The dot product of this matrix with its transpose yielded a 20 × 20 co-occurrence matrix representing the number of sensations in which each pair of descriptors co-occurred (Salton and McGill, 1983; Church and Hanks, 1989). The diagonal was set to zero to remove self-co-occurrences (Everitt et al., 2011). The co-occurrence matrix was then transformed into a condensed distance matrix using Euclidean distances between row vectors (Legendre and Legendre, 2012).

##### 2.7.3.2 Clustering

Hierarchical agglomerative clustering was performed using Ward’s method as implemented in SciPy (Ward, 1963). The optimal number of clusters was determined using the Elbow method (Ketchen Jr and Shook, 1996), based on the reduction in within-cluster variance. Robustness of the clustering solution was evaluated via jackknife resampling, systematically omitting one descriptor at a time and re-running the clustering for k = 2 to 10 clusters (Efron, 1982). Stability was quantified as the proportion of descriptors retaining their original cluster assignment. Cluster centroids were computed as the mean co-occurrence vectors of the descriptors within each cluster.

##### 2.7.3.3 Assigning sensations to clusters

For each sensation, the Jaccard index (intersection size divided by union size) was calculated between its set of descriptors and each cluster’s descriptor set. A sensation was assigned to all clusters whose Jaccard score was ≥ 90% of that sensation’s highest score, allowing multi-cluster assignments when descriptor overlap was nearly equivalent.

##### 2.7.3.4 Cluster distributions

For each descriptor cluster, all binary sensation masks assigned to that cluster were summed at the pixel level, and the resulting overlap counts were normalized by the total number of masks, as described above.

##### 2.7.3.5 Cluster comparisons

Mean pixel intensity, spatial extent (% template surface), proportion of referred sensations, and laterality (ipsilateral, contralateral, bilateral) were calculated for each cluster. Differences across clusters were tested using Kruskal–Wallis tests with post-hoc Dwass–Steel–Critchlow–Fligner pairwise comparisons.

#### 2.7.4 Shape analysis of attention-related referred sensations

To characterize the geometry of attention-related referred sensations in the FSA group, we performed a contour-based shape analysis on all referred sensations assigned to descriptor cluster 4 (see above). The analysis followed a three-stage computational pipeline: (1) image preprocessing and contour extraction, (2) shape feature quantification, and (3) unsupervised clustering and evaluation.

##### 2.7.4.1 Image preprocessing and contour extraction

Sensation maps were converted to 8-bit grayscale and smoothed with a Gaussian kernel (*σ* = 5 px) to reduce high-frequency noise. Binary masks were generated using a global threshold (pixel value ≥ 1). Continuous contours were extracted using OpenCV’s border-following algorithm. Only contours with ≥ 100 pixels were retained for further analysis.

##### 2.7.4.2 Shape feature computation

For each contour, the following geometric features were extracted: (i) Area—number of pixels within the contour; (ii) Perimeter—length of the contour boundary; (iii) Shape ratio—width-to-height ratio of the minimum bounding box [values > 1 = horizontal elongation; values < 1 = vertical elongation (Russ, 2016)]; (iv) Perimeter-to-area ratio—contour perimeter divided by enclosed area [higher values indicate more elongated or fragmented shapes (Da Fontoura Costa and Cesar Jr, 2010)]; (v) Elongation—major-to-minor axis ratio of the best-fitting ellipse [higher values indicate stronger deviation from isotropy (Zunic and Rosin, 2004)].

These features are widely used to characterize morphological variation in image-based perceptual and biological data (Zunic and Rosin, 2004; Da Fontoura Costa and Cesar Jr, 2010; Russ, 2016). They were selected to distinguish elongated, line-like patterns from more compact or irregular regions. Elongated structures were expected to show higher perimeter-to-area ratios and elongation values than compact shapes, along with bounding-box ratios that deviated substantially from unity in accordance with their orientation.

##### 2.7.4.3 Model selection and scope

Given the exploratory nature of this study, model selection proceeded iteratively. We first applied the shape-clustering pipeline to all FSA sensations (local and referred) and observed three recurrent geometric archetypes on inspection of the feature space: point-like/very localized, diffuse/areal, and elongated/pathway-like shapes. Because localized shapes overwhelmingly coincided with pressure/throbbing (local sensations) and our primary interest was referred phenomena, subsequent analyses were restricted to referred sensations within the attention-related descriptor cluster (see above). In this subset, only two families remained—elongated and non-elongated (compact/patch-like). We therefore fit a two-cluster solution on substantive grounds. As the Elbow heuristic is often uninformative at k = 2, we relied on resampling-based stability to justify the solution and verified clear separation on elongation and perimeter-to-area features. The decision to include an elongated class is consistent with our previous work on line-like, pathway-like sensations under sensory deprivation and placebo/attention paradigms (Beissner and Marzolff, 2012; Beissner et al., 2015; Beissner, 2020).

##### 2.7.4.4 Outlier removal

To reduce the influence of extreme cases, shapes with feature values outside the 1st–99th percentile range were excluded prior to clustering (Wilcox, 2012).

##### 2.7.4.5 Clustering of shapes

Standardized feature vectors were clustered using k-means and hierarchical clustering (Jain, 2010). Pairwise scatter plots of all feature combinations (e.g., shape ratio vs. elongation) were generated to illustrate separation between clusters. To assess robustness, clustering solutions were evaluated with resampling-based stability analyses, quantifying the consistency of cluster assignments across repeated subsamples (Lange et al., 2004; Hennig, 2007).

##### 2.7.4.6 Cluster property analysis

For each shape cluster, we calculated mean intensity, extent (% template surface), laterality (ipsilateral, contralateral, bilateral), and complexity (mean number of descriptors per sensation). Spatial distribution maps were generated to visualize the body locations most frequently associated with each cluster.

## 3 Results

### 3.1 Somatic attention produces distinct sensory experiences

When participants deliberately directed attention into their bodies and toward specific body regions, they consistently reported sensations that differed qualitatively and quantitatively from those in natural control conditions.

**Figure 2:**
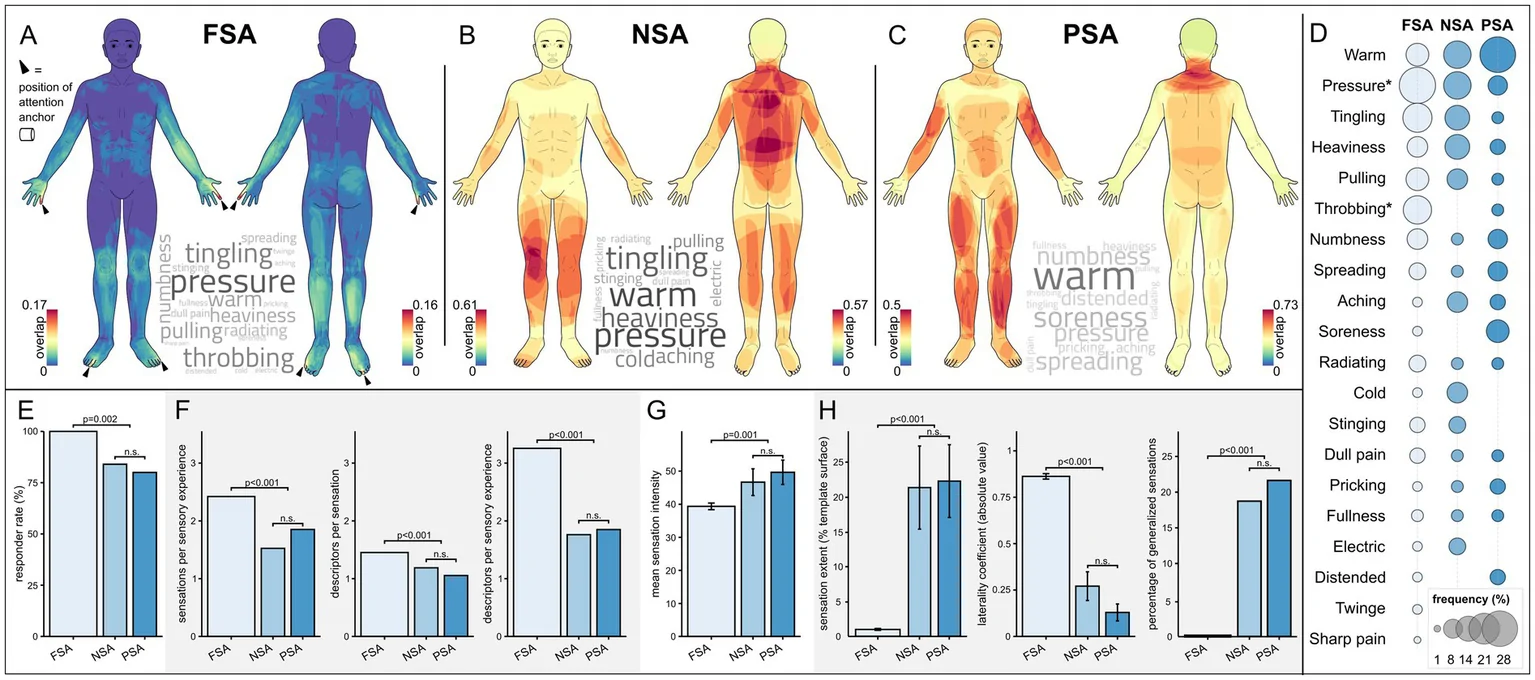
Somatic attention produces distinct sensory experiences. Participants in the three experimental groups reported sensations that differed both qualitatively and quantitatively. **(A–C)** Overlap maps of sensation distributions in the FSA **(A)**, NSA **(B)**, and PSA **(C)** groups. **(A)** FSA participants exhibited highly localized sensations focused on the extremities, with the greatest overlap at the sites of the attention anchors and some centripetal spread. Almost no sensations were reported for the trunk or head region. In contrast, both the NSA **(B)** and PSA group **(C)** showed much broader, less focal distributions: Overlap values were substantially higher in these groups, indicating diffuse sensations primarily concentrated in the back and neck regions, with additional involvement of the arms and legs (all maps thresholded at ≥ 2 sensations). **(D)** Experimental groups also differed in their descriptor profiles with significant group differences for “pressure” and “throbbing.” The FSA and PSA group bore some similarities regarding their most frequently reported descriptors (“warm,” “pressure,” “tingling,” “pulling,” and “heaviness”), while the PSA group primarily reported “warm” sensations. **(E)** The responder rate was higher in the FSA group as compared to NSA and PSA, which showed no significant differences. **(F)** Sensory experiences of FSA participants were more complex, as evidenced by a higher number of sensations and descriptors per sensory experience and more descriptors per sensation. **(G)** In contrast, mean sensation intensity was significantly higher in the NC groups. **(H)** Sensation extent was considerably lower in the FSA group as compared to NC with sensations being more lateralized. Generalized sensations (i.e., those covering more than two thirds of the body template) were observed only in NC participants.

Spatial distributions differed markedly between groups. FSA sensations were mostly localized to anchor sites on the extremities, with only occasional centripetal spread toward proximal limb segments. Sensations in the trunk and head were rare (Figure 2A). By contrast, sensations in the control groups were diffuse, with high overlap in the back and neck regions and additional coverage of arms and legs (Figures 2B,C).

The qualitative profiles also diverged. FSA participants most frequently reported “pressure” (28%), “tingling” (19%), and “throbbing” (17%), while controls more often reported “warm” (22%), “pressure” (12%) and “heaviness” (9%) (Figure 2D). Notably, “pressure” and “throbbing” were significantly enriched in FSA compared to controls (“pressure”: OR = 2.97, 95% CI [1.38–6.36], Fisher’s exact test, two-tailed, raw *p* = 0.004, *q* = 0.024; “throbbing”: OR = 13.5, 95% CI [1.85–98.9], raw *p* < 0.001, *q* = 0.003).

All FSA participants reported sensations in every trial, whereas responder rates were significantly lower in both control groups (Fisher’s exact test, OR = ∞, *p* ≈ 0.002; Table 1, Figure 2E). FSA sensations were more numerous and complex, with participants reporting a higher number of sensations per trial (median = 2.25, IQR = [1.81, 2.75] vs. median = 1, IQR = [1,2]; Mann–Whitney *U* = 496, *n*₁ = 50, *n*₂ = 41, *p* < 0.001, rank-biserial correlation = 0.516) and more descriptors per sensation (median = 1, IQR = [1, 2] vs. median = 1, IQR = [1,1]; Mann–Whitney *U* = 12,853, *n*₁ = 482, *n*₂ = 69, *p* < 0.001, rank-biserial correlation = 0.227; Table 1, Figure 2F). The repertoire of descriptors was also broader, with FSA participants drawing on a larger set of unique terms.

**Table 1:** Characteristics of reported sensations across the Focused Somatic Attention (FSA), Natural Somatic Attention (NSA), and Passive Somatic Awareness (PSA) groups.

Measure	FSA (*n* = 50)	NSA (*n* = 25)	PSA (*n* = 25)	NC (*n* = 50)	FSA vs NC
Participants, *n*	50	25	25	50	–
Responders, *n* (%)	50 (100%)	21 (84%)	20 (80%)	41 (82%)	Fisher, OR = ∞, *p* ≈ 0.002
Sensations analyzed, *n*	482	36	42	78	–
Sensations per experience, median [IQR]	2.25 [1.81–2.75]	1.00 [1.00–2.00]	1.00 [1.00–2.00]*p* <	1.00 [1.00–2.00]	*U* = 496, *p* < 0.001, *r* = 0.52
Descriptors per sensation, median [IQR]	1 [1–2]	1 [1–1]	1 [1–1]	1 [1–1]	*U* = 12,853, *p* < 0.001, *r* = 0.23
Sensation intensity (0–100), median [IQR]	34.6 [22.4–52.9]	46.4 [33.9–54.0]	48.2 [36.4–64.3]	46.8 [34.4–58.6]	*U* = 12,611, p = 0.001, *r* = −0.24
Sensation extent (% template), median [IQR]	0.22 [0.04–0.9]	3.42 [0.97–17.85]	3.75 [1.39–24.99]	3.69 [1.16–21.9]	*U* = 8,202, *p* < 0.001, *r* = −0.56
Generalized (whole-body) sensations, *n* (%)	0 (0%)	6 (17%)	8 (19%)	14 (18%)	Fisher, OR = ∞, *p* < 0.001

Continuous measures use the Mann–Whitney *U* test with the rank-biserial correlation r as effect size, and responder rate and generalized sensations use Fisher’s exact test. Sensations per experience are the per-trial counts. Sensation intensity is the mean masked drawing intensity rescaled to 0–100. Sensation extent is the percentage of the body template covered by the sensation. Referred-versus-local classification and spatial-morphology analyses are reported separately, as they apply to the FSA group only.

In contrast, mean sensation intensity was lower in FSA than in controls (median = 34.6, IQR = [22.4, 52.9] vs. median = 46.8, IQR = [34.4, 58.6]; Mann–Whitney *U* = 12,611, *n*₁ = 482, *n*₂ = 69, *p* = 0.001, rank-biserial correlation = −0.242; Table 1, Figure 2G). Sensation extent was markedly smaller in FSA compared to controls (median = 0.22 percent of template surface, IQR = [0.04, 0.9] vs. median = 3.69 percent, IQR = [1.16, 21.9]; Mann–Whitney *U* = 8,202, *n*₁ = 482, *n*₂ = 78, *p* < 0.001, rank-biserial correlation = −0.564) and generalized sensations covering most of the body occurred only in controls (Fisher’s exact test, OR = ∞, *p* < 0.001; Table 1, Figure 2H). Moreover, FSA sensations were strongly lateralized, predominantly ipsilateral to the attended side, whereas control sensations were often bilateral and non-specific (Figure 2H).

Together, these findings demonstrate that focused somatic attention gives rise to a distinct class of bodily experiences: frequent, complex, spatially structured, and lateralized, yet of lower intensity and extent than naturally arising sensations.

### 3.2 A new class of referred, pathway-like sensations

**Figure 3:**
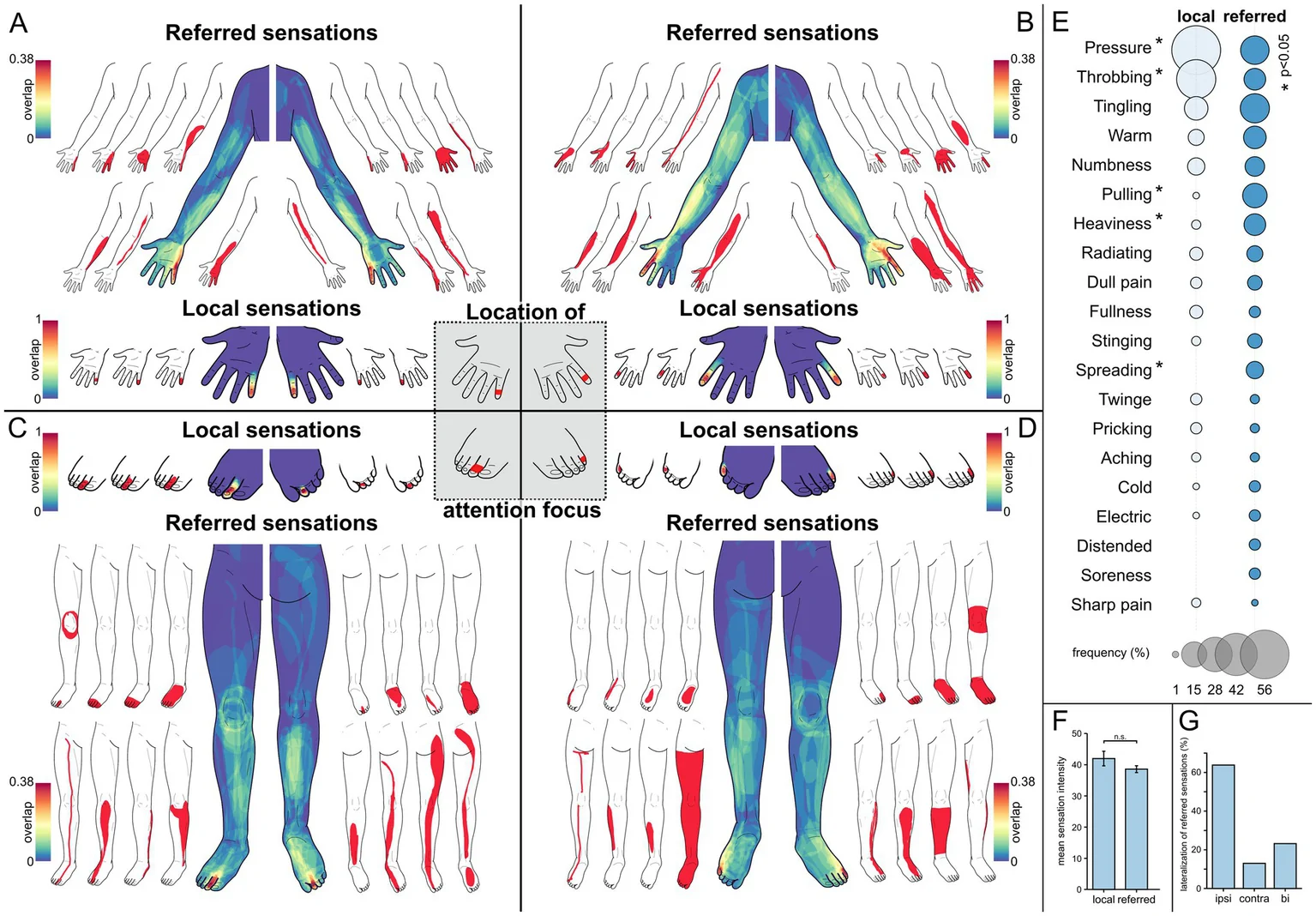
A new class of referred, pathway-like sensations **(A–D)** Group-level overlap maps and representative individual drawings (red) of local and referred sensations across the four anchor sites (A: right little finger, B: left index finger, C: right second toe, D: left little toe). All maps thresholded at ≥ 2 sensations. Local sensations were almost exclusively confined to the anchor region and rarely extended beyond the immediate finger or toe mask. Referred sensations occurred at distant body sites, sometimes including the anchor region but often appearing entirely away from it. Distributions were either diffuse or elongated up to line-like, forming linear pathways across multiple joints. Overlap values are normalized to the total number of masks per category. **(E)** Descriptor profiles for local and referred sensations. Local sensations (*n* = 112) were most frequently described as pressure (56.3%), throbbing (35.7%), and tingling (12.5%). Referred sensations (*n* = 370) were characterized by tingling (20.3%), pressure (19.5%), pulling (13.5%), warm (12.2%), and throbbing (10.8%). Statistical comparisons confirmed that pressure and throbbing were significantly more common in local sensations, while heaviness, spreading, and pulling were more common in referred sensations (FDR-corrected Fisher’s exact tests). **(F)** Sensation intensity did not differ significantly between local and referred sensations. **(G)** Distribution of side dominance categories for referred sensations. While local sensations were, by definition, ipsilateral, referred sensations included ipsilateral, contralateral, and bilateral cases with the great majority being ipsilateral.

The majority of sensations generated by focused somatic attention were not confined to the site of attentional focus. Across all trials, 76.8% of FSA sensations were referred to locations remote from the stimulated finger or toe (*n* = 370 of 482), whereas only 23.2% were local (*n* = 112; *χ*^2^(1) = 276.20, *p* < 0.001, Cramer’s *V* = 0.535). On the individual level, 94% of participants reported referred sensations, and most did so for multiple anchor sites. Thirty-five participants experienced referred sensations for all four anchor locations (Figures 3A–D).

Referred sensations were striking in form. Whereas local sensations were confined to the anchor region and most often described as “pressure” or “throbbing,” referred sensations frequently appeared at distant sites, sometimes entirely away from the anchor. Their distributions ranged from diffuse patches to elongated pathway-like shapes spanning multiple joints (Figures 3A–D). Descriptor usage distinguished the two categories: “pressure” (56%) and “throbbing” (36%) dominated local reports (pressure: OR = 0.188, 95% CI [0.119–0.296], *χ*^2^(1) = 57.7; raw *p* < 0.001, *q* < 0.001; throbbing: OR = 0.218, 95% CI [0.131–0.362], *χ*^2^(1) = 38.5, raw *p* < 0.001, *q* < 0.001), while “pulling” (14%), “heaviness” (11%), and “spreading” (7%) were significantly enriched in referred experiences (pulling: OR = 17.3, 95% CI [2.37–127], *χ*^2^(1) = 14.5, raw *p* < 0.001, *q* < 0.001; heaviness: OR = 6.48, 95% CI [1.54–27.3], *χ*^2^(1) = 8.47, raw *p* = 0.004, *q* = 0.007; spreading: OR = 18.0, 95% CI [1.09–298], *χ*^2^(1) = 8.66, raw *p* = 0.003, *q* = 0.007; FDR-corrected; Figure 3E).

Sensation intensity did not differ between local and referred categories (median = 34.5, IQR = [22.4–55.9] vs. median = 34.6, IQR = [22.2–52.1]; Mann–Whitney *U* = 19,548, *n*₁ = 112, *n*₂ = 370, *p* = 0.364, rank-biserial correlation = −0.0566; Figure 3F).

Although local sensations were by definition ipsilateral, referred sensations included ipsilateral (64%), contralateral (13%), and bilateral (23%) cases (Figure 3G).

These results show that focused somatic attention generates a new class of bodily sensations characterized by referral over long distances and pathway-like spatial distributions across the body.

### 3.3 Qualitative organization of sensation descriptors

**Figure 4:**
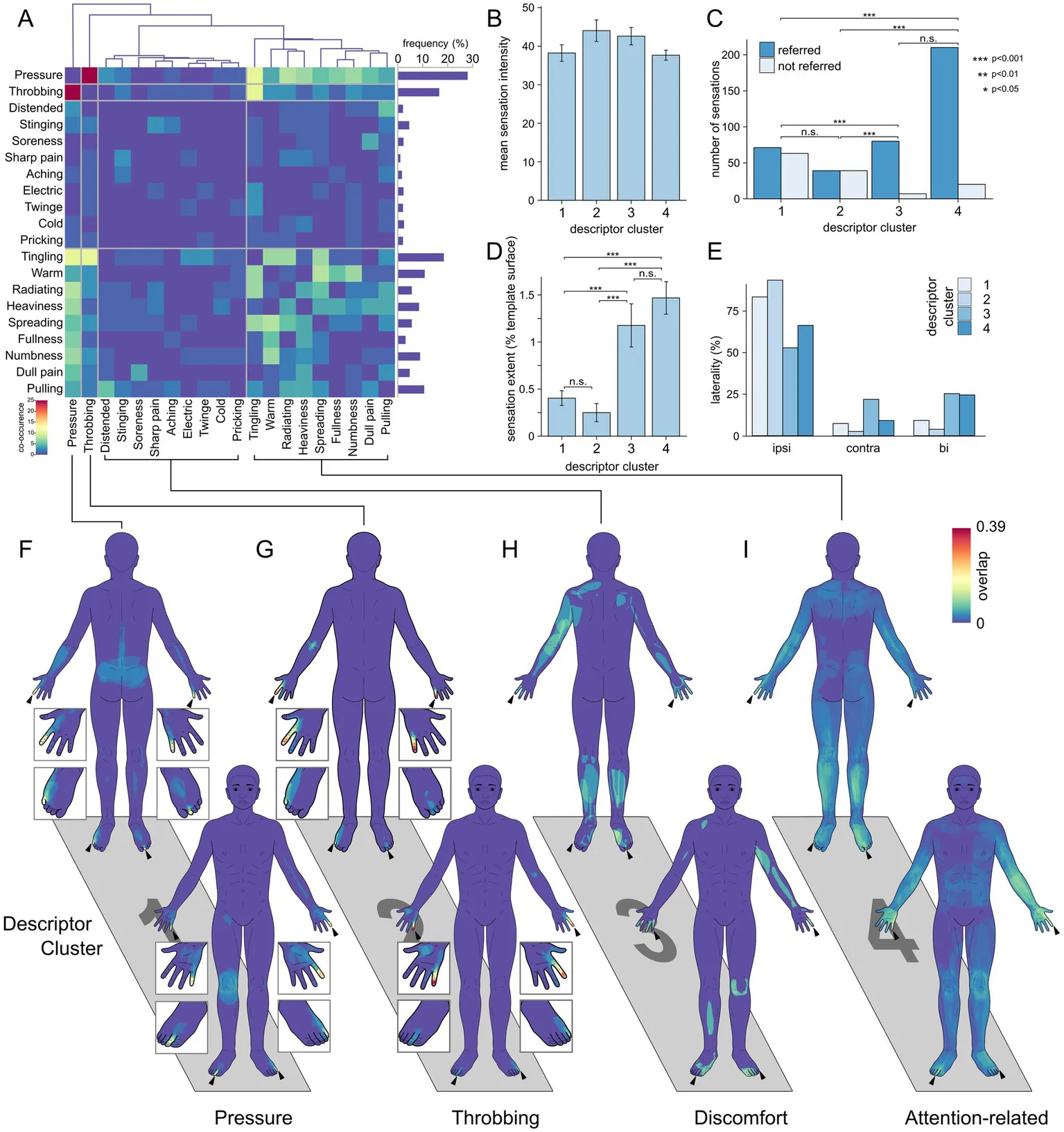
Qualitative organization of sensation descriptors. **(A)** Hierarchical clustering of the descriptor co-occurrence matrix revealed four clusters. Clusters 1 and 2 consisted of single, high-frequency descriptors (pressure; throbbing), while Cluster 3 (aching, stinging, soreness, pricking, sharp pain, electric, twinge, cold, distended) was characterized by low frequency and weak co-occurrence. Cluster 4 (tingling, warm, radiating, heaviness, spreading, fullness, numbness, dull pain, pulling) showed moderate frequencies and strong internal co-occurrence. **(B–E)** Spatial distributions. Clusters 1 and 2 were confined to the anchor region **(B–C)**. Cluster 3 descriptors were more variable and widespread without a consistent pattern **(D)**. Cluster 4 was strongly associated with referred sensations and showed diffuse or elongated patterns extending across multiple joints **(E)**. All maps thresholded at ≥ 2 sensations. **(F–I)** Quantitative comparisons across clusters. Intensity did not differ significantly **(F)**, while differences emerged for the laterality **(G)**, spatial extent **(H)**, and proportion of referred sensations **(I)**.

To examine whether these novel sensations were associated with distinct qualitative profiles, we analyzed descriptor co-occurrence in the FSA group. Four robust descriptor clusters emerged (Figure 4A): Clusters 1 and 2 consisted of single, high-frequency descriptors (pressure; throbbing), while Cluster 3 (aching, stinging, soreness, pricking, sharp pain, electric, twinge, cold, distended) was characterized by low frequency and weak co-occurrence. Finally, Cluster 4 (tingling, warm, radiating, heaviness, spreading, fullness, numbness, dull pain, pulling) showed moderate frequencies and strong internal co-occurrence. Furthermore, Clusters 1 and 2 co-occurred frequently with each other but differed in their association with Cluster 4 in that Cluster 1 had stronger links to Cluster 4 compared to Cluster 2.

These descriptor clusters corresponded to distinct bodily distributions (Figures 4B–E). Clusters 1 and 2 were largely confined to anchor regions. In contrast, Cluster 4 was strongly associated with spreading, referred sensations often forming elongated patterns extending across multiple joints. Cluster 3 was more variable and its localization less consistent.

Quantitative comparisons supported these findings. While sensation intensity was comparable accross clusters (*χ*^2^(3) = 6.38, *p* = 0.094; Figure 4F), the proportion of referred sensations differed significantly between clusters (*χ*^2^(3) = 108.1, *p* < 0.001, *ε*^2^ = 0.205; Figure 4G): Clusters 1 and 2 were predominantly local, whereas Clusters 3 and 4 were largely referred.

Sensation extent also varied (*χ*^2^(3) = 119.6, *p* < 0.001, *ε*^2^ = 0.227), with sensations of Clusters 3 and 4 (median = 0.33% template surface, IQR = [0.09–1.39] and median = 0.54%, IQR = [0.19–1.59]) covering larger areas than those of Clusters 1 and 2 (median = 0.05% template surface, IQR = [0.03–0.28] and median = 0.05%, IQR = [0.03–0.09]; Figure 4H). Laterality differed as well (*χ*^2^(6) = 58.2, *p* < 0.001, Cramer’s *V* = 0.234), with Clusters 1 and 2 more strictly ipsilateral, while Clusters 3 and 4 were more variable (Figure 4I). These results show that the new class of referred sensations aligns with a distinct qualitative family of descriptors.

### 3.4 Shape analysis reveals compact and elongated categories

**Figure 5:**
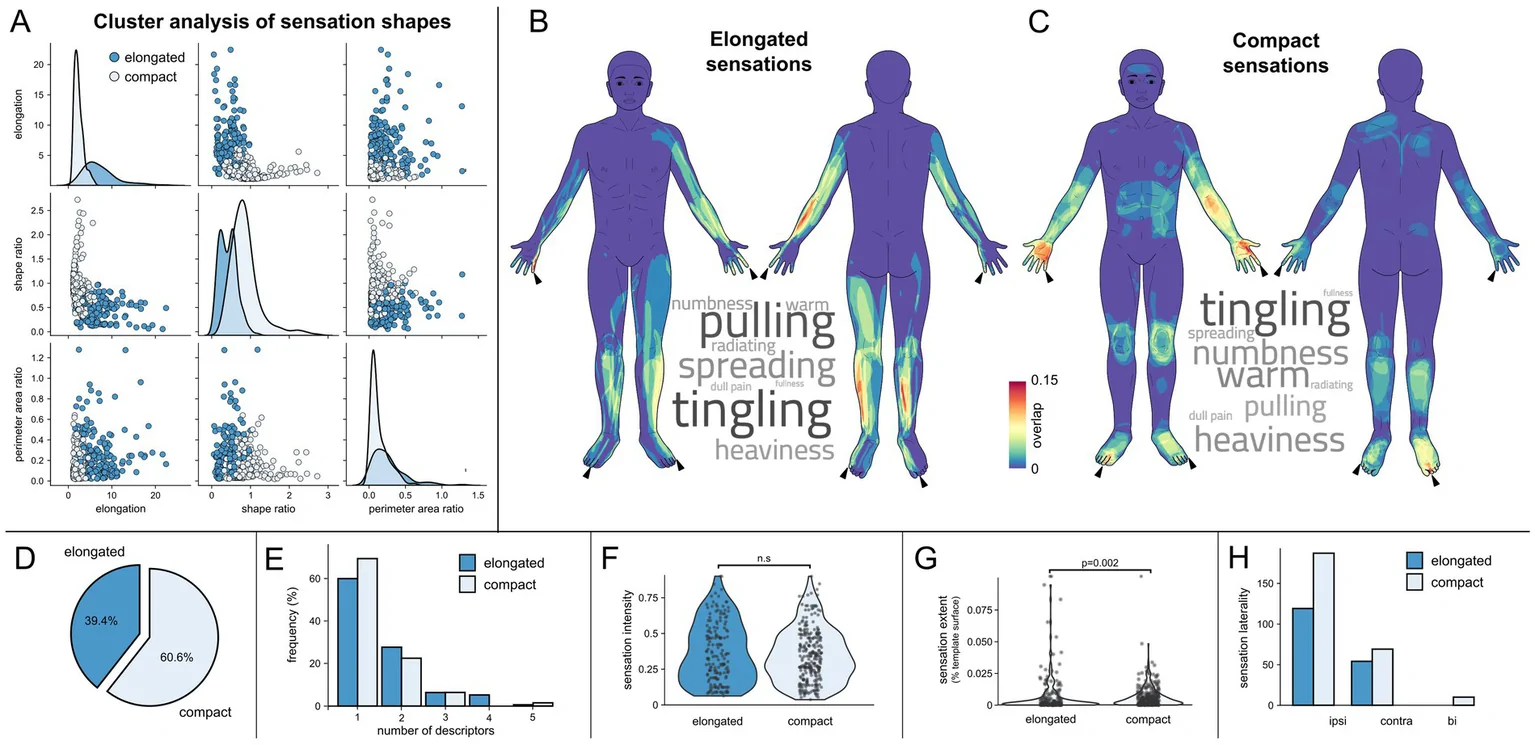
Shape analysis reveals compact and elongated categories. **(A)** Clustering of shape features revealed two categories: Compact (irregular) and elongated (linear) shapes. For representative individual drawings see Supplementary Figure 2. **(B, C)** Cluster-based overlap maps and corresponding descriptor word clouds. Elongated shapes were found exclusively on the extremities, tracing linear pathways across multiple joints **(B)**, whereas compact shapes appeared at distal extremities as well as on the abdomen, upper back, and knees (C; all maps thresholded at ≥ 2 sensations). **(D)** Cluster proportions: Compact shapes (*n* = 266) occurred significantly more often than elongated shapes (*n* = 173, *p* < 0.001). **(E)** Mean sensation intensity did not differ significantly between clusters. **(F)** Compact shapes covered a significantly larger proportion of the body template than elongated shapes. **(G)** Laterality distributions: Both clusters were predominantly ipsilateral, but compact shapes showed more bilateral cases, whereas elongated shapes included slightly more contralateral cases. **(H)** Descriptor complexity: Elongated shapes were characterized by a higher number of descriptors per sensation, indicating greater complexity.

To characterize the geometry of attention-related referred sensations, we applied a contour-based clustering analysis to digital drawings. Two geometric categories emerged (Figure 5A): compact (and irregular) shapes (*n* = 266, 61%) and elongated shapes (*n* = 173, 39%). Compact shapes were patches of limited extent, while elongated shapes traced linear, pathway-like forms across multiple joints (Figures 5B,C).

Compact shapes were more frequent (*χ*^2^(1) = 19.7, *p* < 0.001; Figure 5D), comparable in sensation intensity (*t*(437) = 1.23, *p* = 0.221; Figure 5E) and exhibited greater spatial extent than elongated shapes (median = 0.38% template surface, IQR = [0.09–0.87] vs. median = 0.17%, IQR = [0.04–0.71]; Mann–Whitney *U* = 18,955, *n*₁ = 173, *n*₂ = 266, *p* = 0.0018, rank-biserial correlation = 0.176; Figure 5F). Both categories were predominantly ipsilateral, but elongated shapes included slightly more contralateral cases (31% vs. 26%, Fisher’s exact test, two-tailed, *p* = 0.013, Cramer’s *V* = 0.131, Figure 5G). In contrast, elongated sensations showed a slightly higher descriptor complexity (median = 1, IQR = [1–2] vs. median = 1, IQR = [1–2]; Mann–Whitney *U* = 20,591, *n*₁ = 173, *n*₂ = 266, *p* = 0.026, rank-biserial correlation = −0.105; Figure 5H).

These findings confirm that attention-related referred sensations are not homogeneous but instead fall into two categories: compact patches and elongated, pathway-like experiences. The elongated category is less frequent and distinguished by greater complexity and smaller extent. It represents the clearest manifestation of a new class of bodily sensation generated by focused somatic attention.

## 4 Discussion

Our findings demonstrate that deliberately directing attention into the body—somatic attention—and focused on a lightly touched body region, generates structured, pathway-like bodily sensations. Although a minimal tactile anchor was present at the point of focus, the sensations that arose were predominantly referred to distant body regions that received no external input, and were often elongated across multiple joints. Rather than simply amplifying or suppressing pre-existing inputs, attention here acted as a constructive force, giving rise to experience at sites where nothing was stimulated. These results reframe attention as generative, not merely modulatory, in shaping human bodily experience.

This conceptual shift resonates with predictive coding theories of perception, which maintain that sensory experience is a mixture of both ascending input and top–down predictions (Friston, 2005; Clark, 2013). Under restricted environmental stimulation, bottom-up input may be so minimal, that the balance tilts toward top–down contributions, thus allowing attention to build coherent percepts even without pre-existing sensations. Focused somatic attention may thus function analogously to placebo stimulation, where expectation generates sensory qualities such as numbness or warmth (Salih et al., 2010; Beissner et al., 2015). Consistent with this interpretation, a large body of research shows that attention and expectation modulate pain perception. Directing attention toward or away from nociceptive stimuli alters both perceived intensity and neural processing of pain signals, highlighting the role of top–down influences in shaping bodily experience (Tracey et al., 2002; Villemure and Bushnell, 2002).

This mechanistic account can be situated within a broader functional framework. On an allostasis-first view of brain function (Theriault et al., 2025), predictive processing ultimately serves the anticipatory regulation of the body, and interoception is one expression of this regulatory modelling. Restricted environmental stimulation is, from this perspective, not a state of inactivity but one of reduced exteroceptive competition, in which interoceptive and somatosensory predictions continue and may be weighted more heavily (Ainley et al., 2016). Our findings therefore need not reflect a simple reduction of bottom-up input with greater top-down weighting; they may instead reflect a shift in the composition of ongoing signals, with internally generated bodily predictions contributing relatively more to perception. While we did not measure interoceptive or visceral signals and cannot attribute the observed sensations to allostatic regulation directly, this framing offers a parsimonious account of why focused inward attention, under reduced exteroceptive load, might render bodily predictions perceptible and spatially organized, thus complementing, rather than replacing, the generative interpretation above.

Our results provide new insights into the broader phenomenon of referred sensations, and referred pain in particular (Head, 1893; Feinstein et al., 1954; Katz and Melzack, 1987). Classical studies have shown that sensations can be displaced beyond their origin, yet the underlying mechanism remains unresolved. The prevailing convergence–projection model attributes referral to spinal neurons receiving input from multiple sites, while work on central sensitization emphasizes the enlargement of receptive fields during sustained nociceptive input (Mense, 1994; Arendt-Nielsen and Svensson, 2001). Neuroimaging has further elucidated potential cortical mechanisms: While the primary somatosensory cortex encodes body regions in a topographic manner, higher-order regions in the parietal and insular cortex integrate these maps into coherent bodily representations (Kaas, 1997). Prior studies on spontaneous and attention-related sensations similarly implicate a cortical–hippocampal–insular network (Bauer et al., 2014). We propose that focused attention amplifies activity at focal points, which then propagates along experientially connected representations until a coherent pathway emerges. Such dynamics resemble what we know from multisensory illusions like the rubber hand (Ehrsson et al., 2004), the cutaneous rabbit (Blankenburg et al., 2006), or projection phenomena where sensations extend to external objects (Armel and Ramachandran, 2003). Taken together with classical evidence on referred pain, these findings support the view that focused somatic attention can recruit existing body maps to generate novel, spatially organized percepts.

Somatic attention in general but also its focused form is a central component of a broad class of practices that deliberately cultivate heightened bodily awareness. In mind–body practices and contemplative traditions such as mindfulness meditation, qigong, and tai chi, practitioners direct attention inward to deepen awareness of internal states and processes (Mehling et al., 2011). Sensations closely resembling those observed in our study (tingling, warmth, spreading, or energy-like currents) are frequently reported in these contexts (Beissner, 2020; Cooper et al., 2021). Our findings indicate that such sensations are not dependent on cultural frameworks, suggestion, or extensive training, but can emerge spontaneously under the right circumstances when attention is directed into the body. Restricted environmental stimulation could be the decisive factor in creating a physical-mental state that would otherwise only be achieved through contemplative practice, albeit in a minimal and experimentally controlled form.

Another interesting finding in this context are the elongated or pathway-like sensation shapes reported by our participants. These are reminiscent of long-standing cultural constructs of bodily pathways, such as the meridians described in classical Chinese sources (Unschuld, 1985) or the nadis described in South Asian yogic traditions (Mallinson and Singleton, 2017), as well as similar notions in other cultures. While our findings do not validate these models, they demonstrate that pathway-like bodily experiences are experimentally reproducible and organized rather than random. By providing a minimal experiment in which focused attention alone is sufficient to generate such experiences, our study points to a generic mechanism that may underlie the cross-cultural and historical ubiquity of these sensations.

Our study has several limitations. First, our evidence rests on subjective reports and behavioural measures rather than direct neural recordings; no neuroimaging or electrophysiology was employed. This was a deliberate scope decision, as establishing and quantitatively characterising the behavioural phenomenon is a necessary precondition for any subsequent mechanistic study, and we reduced the reliance on introspection by capturing the sensations objectively through validated digital sensation mapping. Nonetheless, the absence of a neural measure means that, while the referred, remote character of the sensations is difficult to attribute to peripheral input, we cannot directly demonstrate that they are internally generated; objective physiological or neural measures would provide this confirmation, and we regard neuroimaging as the clear next step (see Future directions). We further note that recording such signals within a floatation tank under restricted environmental stimulation is itself methodologically demanding (water immersion, electrical noise, and movement artefacts). Second, bodily experiences are phenomenologically much richer than could be captured using quantitative assessments. Furthermore, our categorical analyses may not encompass all subtleties. Third, we observed considerable variability across individuals. This suggests that susceptibility to somatic attention differs depending on cognitive style or prior training of the individual, as proposed in earlier studies (Mehling, 2016). Finally, our participants were healthy adults and the FSA group was younger than controls, limiting generalizability to other populations.

### 4.1 Conclusion

In summary, focused somatic attention directed toward a minimal, localized tactile anchor under restricted environmental stimulation gives rise to a qualitatively new class of bodily experience: frequent, structured, and predominantly referred sensations that are spatially organized into compact patches and elongated, pathway-like forms spanning multiple joints. These experiences are distinct from those arising under natural somatic attention or passive awareness, and their non-random qualitative and spatial regularities indicate that attention can contribute generatively, and not merely modulatorily, to somatosensory experience. The paradigm thus offers a tractable psychophysical model for studying top-down contributions to body representation and to referred sensation. More broadly, our findings suggest that the felt body is not a fixed substrate but a dynamic construct, continually redefined by attentional processes.

### 4.2 Future directions

Several directions follow from this work. Because the present study is behavioural, neuroimaging (e.g., fMRI or MEG) is the natural next step to identify the neural substrates that generate these sensations and to test the predictive-coding account directly, for example by manipulating expectation. The paradigm should also be replicated in age-balanced samples and extended across anchor types and sites, durations, and instructions, to disentangle the respective contributions of focused attention and low-level input. Finally, given their resemblance to clinically relevant phenomena, attention-generated referred sensations may provide an experimental window onto maladaptive bodily experiences, for example in chronic pain (Moseley and Flor, 2012; Denk et al., 2014) or anxiety disorders (Paulus and Stein, 2010). Clinically, training somatic attention could help reshape such experiences and offer a non-pharmacological means of deliberately modulating body representation.

## Data Availability

The datasets presented in this study can be found in online repositories. The names of the repository/repositories and accession number(s) can be found at: https://doi.org/10.5281/zenodo.17054829.

## References

[ref1] Ainley V. Apps M. A. J. Fotopoulou A. Tsakiris M. (2016). “Bodily precision”: a predictive coding account of individual differences in interoceptive accuracy. Philos. Trans. R. Soc. Lond. Ser. B Biol. Sci. 371:20160003. doi: 10.1098/rstb.2016.0003, 28080962 PMC5062093

[ref2] Andersen L. M. Lundqvist D. (2019). Somatosensory responses to nothing: an MEG study of expectations during omission of tactile stimulations. NeuroImage 184, 78–89. doi: 10.1016/j.neuroimage.2018.09.014, 30213774

[ref3] Arendt-Nielsen L. Svensson P. (2001). Referred muscle pain: basic and clinical findings. Clin. J. Pain 17, 11–19. doi: 10.1097/00002508-200103000-00003, 11289083

[ref4] Armel K. C. Ramachandran V. S. (2003). Projecting sensations to external objects: evidence from skin conductance response. Proc. R. Soc. Lond. B 270, 1499–1506. doi: 10.1098/rspb.2003.2364, 12965016 PMC1691405

[ref5] Bauer C. C. C. Barrios F. A. Díaz J.-L. (2014). Subjective somatosensory experiences disclosed by focused attention: cortical-hippocampal-insular and amygdala contributions. PLoS One 9:e104721. doi: 10.1371/journal.pone.0104721, 25166875 PMC4148258

[ref6] Beissner F. (2020). Therapeutic sensations: a new unifying concept. Evid. Based Complement. Alternat. Med. 2020:7630190. doi: 10.1155/2020/7630190, 32831879 PMC7428881

[ref7] Beissner F. Brünner F. Fink M. Meissner K. Kaptchuk T. J. Napadow V. (2015). Placebo-induced somatic sensations: a multi-modal study of three different placebo interventions. PLoS One 10:e0124808. doi: 10.1371/journal.pone.0124808, 25901350 PMC4406515

[ref8] Beissner F. Marzolff I. (2012). Investigation of acupuncture sensation patterns under sensory deprivation using a geographic information system. Evid. Based Complement. Alternat. Med. 2012, 1–10. doi: 10.1155/2012/591304, 23243458 PMC3518766

[ref9] Blankenburg F. Ruff C. C. Deichmann R. Rees G. Driver J. (2006). The cutaneous rabbit illusion affects human primary sensory cortex Somatotopically. PLoS Biol. 4:e69. doi: 10.1371/journal.pbio.0040069, 16494530 PMC1382015

[ref10] Carrasco M. (2011). Visual attention: the past 25 years. Vis. Res. 51, 1484–1525. doi: 10.1016/j.visres.2011.04.012, 21549742 PMC3390154

[ref11] Church K. W. Hanks P. (1989). Word association norms, mutual information, and lexicography. Proceedings of the 27th Annual Meeting on Association for Computational Linguistics. Vancouver: Association for Computational Linguistics, p. 76–83

[ref12] Clark A. (2013). Whatever next? Predictive brains, situated agents, and the future of cognitive science. Behav. Brain Sci. 36, 181–204. doi: 10.1017/S0140525X12000477, 23663408

[ref13] Cooper D. J. Lindahl J. R. Palitsky R. Britton W. B. (2021). “Like a vibration cascading through the body”: energy-like somatic experiences reported by Western Buddhist meditators. Religion 12:1042. doi: 10.3390/rel12121042

[ref14] Critchley H. D. Wiens S. Rotshtein P. Öhman A. Dolan R. J. (2004). Neural systems supporting interoceptive awareness. Nat. Neurosci. 7, 189–195. doi: 10.1038/nn1176, 14730305

[ref15] Da Fontoura Costa L. Cesar R. M. Jr. (2010). Shape Analysis and Classification: Theory and Practice. 0th Edn Boca Raton: CRC Press.

[ref16] De Lange F. P. Heilbron M. Kok P. (2018). How do expectations shape perception? Trends Cogn. Sci. 22, 764–779. doi: 10.1016/j.tics.2018.06.002, 30122170

[ref17] Denk F. McMahon S. B. Tracey I. (2014). Pain vulnerability: a neurobiological perspective. Nat. Neurosci. 17, 192–200. doi: 10.1038/nn.3628, 24473267

[ref18] Desimone R. Duncan J. (1995). Neural mechanisms of selective visual attention. Annu. Rev. Neurosci. 18, 193–222. doi: 10.1146/annurev.ne.18.030195.001205, 7605061

[ref19] Efron B. (1982). The Jackknife, the Bootstrap and other Resampling Plans. Philadelphia, PA: Society for Industrial and Applied Mathematics.

[ref20] Ehrsson H. H. Spence C. Passingham R. E. (2004). That’s my hand! Activity in premotor cortex reflects feeling of ownership of a limb. Science 305, 875–877. doi: 10.1126/science.1097011, 15232072

[ref21] Everitt B. S. Landau S. Leese M. Stahl D. (2011). Cluster Analysis. 1st Edn Hoboken: Wiley.

[ref22] Feinstein J. S. Khalsa S. S. Yeh H. Al Zoubi O. Arevian A. C. Wohlrab C. . (2018). The elicitation of relaxation and interoceptive awareness using floatation therapy in individuals with high anxiety sensitivity. Biol. Psychiatry Cogn. Neurosci. Neuroimaging 3, 555–562. doi: 10.1016/j.bpsc.2018.02.005, 29656950 PMC6040829

[ref23] Feinstein B. Langton J. N. K. Jameson R. M. Schiller F. (1954). Experiments on pain referred from deep somatic tissues. J. Bone Joint Surg. 36, 981–997. doi: 10.2106/00004623-195436050-00007, 13211692

[ref24] Feldman H. Friston K. J. (2010). Attention, uncertainty, and free-energy. Front. Hum. Neurosci. 4:215. doi: 10.3389/fnhum.2010.00215, 21160551 PMC3001758

[ref25] Friston K. (2005). A theory of cortical responses. Philos. Trans. R. Soc. B 360, 815–836. doi: 10.1098/rstb.2005.1622, 15937014 PMC1569488

[ref26] Garfinkel S. N. Critchley H. D. (2013). Interoception, emotion and brain: new insights link internal physiology to social behaviour. Commentary on: “Anterior insular cortex mediates bodily sensibility and social anxiety” by Terasawa et al.(2012). Soc. Cogn. Affect. Neurosci. 8, 231–234. doi: 10.1093/scan/nss140 23482658 PMC3594730

[ref27] Head H. (1893). On disturbances of sensation with especial reference to the pain of visceral disease. Brain 16, 1–133. doi: 10.1093/brain/16.1-2.1

[ref28] Hennig C. (2007). Cluster-wise assessment of cluster stability. Comput. Stat. Data Anal. 52, 258–271. doi: 10.1016/j.csda.2006.11.025

[ref29] Jain A. K. (2010). Data clustering: 50 years beyond K-means. Pattern Recogn. Lett. 31, 651–666. doi: 10.1016/j.patrec.2009.09.011

[ref30] Johansson R. S. Vallbo Å. B. Westling G. (1980). Thresholds of mechanosensitive afferents in the human hand as measured with von Frey hairs. Brain Res. 184, 343–351. doi: 10.1016/0006-8993(80)90803-3, 7353160

[ref31] Kaas J. H. (1997). Topographic maps are fundamental to sensory processing. Brain Res. Bull. 44, 107–112. doi: 10.1016/S0361-9230(97)00094-4, 9292198

[ref32] Katz J. Melzack R. (1987). Referred sensations in chronic pain patients. Pain 28, 51–59. doi: 10.1016/0304-3959(87)91059-1 3822494

[ref33] Ketchen D. J. Jr. Shook C. L. (1996). The application of cluster analysis in strategic management research: an analysis and critique. Strat. Mgmt. J. 17, 441–458. doi: 10.1002/(SICI)1097-0266(199606)17:6<441::AID-SMJ819>3.0.CO;2-G

[ref34] Kilteni K. Ehrsson H. H. (2020). Functional connectivity between the cerebellum and somatosensory areas implements the attenuation of self-generated touch. J. Neurosci. 40, 894–906. doi: 10.1523/JNEUROSCI.1732-19.2019, 31811029 PMC6975290

[ref35] Lange T. Roth V. Braun M. L. Buhmann J. M. (2004). Stability-based validation of clustering solutions. Neural Comput. 16, 1299–1323. doi: 10.1162/089976604773717621, 15130251

[ref36] Legendre P. Legendre L. (2012). Numerical Ecology. Hoboken: Elsevier.

[ref37] Lilly J. C. (1977). The Deep Self: Profound Relaxation and the Tank Isolation Technique. New York: Simon and Schuster.

[ref38] Longo M. R. Schüür F. Kammers M. P. M. Tsakiris M. Haggard P. (2008). What is embodiment? A psychometric approach. Cognition 107, 978–998. doi: 10.1016/j.cognition.2007.12.004 18262508

[ref39] Mallinson J. Singleton M. (2017). Roots of Yoga. London, UK: Penguin Classics.

[ref40] Mehling W. (2016). Differentiating attention styles and regulatory aspects of self-reported interoceptive sensibility. Philos. Trans. R. Soc. B 371:20160013. doi: 10.1098/rstb.2016.0013, 28080970 PMC5062101

[ref41] Mehling W. E. Wrubel J. Daubenmier J. J. Price C. J. Kerr C. E. Silow T. . (2011). Body awareness: a phenomenological inquiry into the common ground of mind-body therapies. Philos. Ethics Humanit. Med. 6:6. doi: 10.1186/1747-5341-6-6, 21473781 PMC3096919

[ref42] Mense S. (1994). Referral of muscle pain. APS J. 3, 1–9. doi: 10.1016/S1058-9139(05)80227-X

[ref43] Moseley G. L. Flor H. (2012). Targeting cortical representations in the treatment of chronic pain: a review. Neurorehabil. Neural Repair 26, 646–652. doi: 10.1177/1545968311433209, 22331213

[ref44] Neubert T. Beissner F. (2019). Hannover body template [dataset]. figshare. doi: 10.6084/m9.figshare.7637387.v9

[ref45] Neubert T.-A. Dusch M. Karst M. Beissner F. (2018). Designing a tablet-based software app for mapping bodily symptoms: usability evaluation and reproducibility analysis. JMIR Mhealth Uhealth 6:e127. doi: 10.2196/mhealth.8409, 29848470 PMC6000481

[ref46] Paulus M. P. Stein M. B. (2010). Interoception in anxiety and depression. Brain Struct. Funct. 214, 451–463. doi: 10.1007/s00429-010-0258-9, 20490545 PMC2886901

[ref47] Posner M. I. Petersen S. E. (1990). The attention system of the human brain. Annu. Rev. Neurosci. 13, 25–42. doi: 10.1146/annurev.ne.13.030190.000325, 2183676

[ref48] Russ J. C. (2016). The image Processing Handbook. Boca Raton: CRC Press.

[ref49] Salih N. Bäumler P. I. Simang M. Irnich D. (2010). Deqi sensations without cutaneous sensory input: results of an RCT. BMC Complement. Altern. Med. 10:81. doi: 10.1186/1472-6882-10-81, 21189142 PMC3022896

[ref50] Salton G. McGill M. J. (1983). Introduction to Modern Information Retrieval. New York: McGraw-Hill.

[ref51] Shaballout N. Neubert T.-A. Boudreau S. Beissner F. (2019). From paper to digital applications of the pain drawing: systematic review of methodological milestones. JMIR Mhealth Uhealth 7:e14569. doi: 10.2196/14569, 31489841 PMC6753689

[ref52] Stein C. Mendl G. (1988). The German counterpart to McGill pain questionnaire. Pain 32, 251–255. doi: 10.1016/0304-3959(88)90074-7, 3362561

[ref53] Storey J. D. Tibshirani R. (2003). Statistical significance for genomewide studies. Proc. Natl. Acad. Sci. USA 100, 9440–9445. doi: 10.1073/pnas.1530509100, 12883005 PMC170937

[ref54] Summerfield C. Egner T. (2009). Expectation (and attention) in visual cognition. Trends Cogn. Sci. 13, 403–409. doi: 10.1016/j.tics.2009.06.003, 19716752

[ref55] The jamovi project (2025). The jamovi project. Available online at: https://www.jamovi.org (Accessed January 21, 2025).

[ref56] Theriault J. E. Katsumi Y. Reimann H. M. Zhang J. Deming P. Dickerson B. C. . (2025). It’s not the thought that counts: Allostasis at the core of brain function. Neuron 113, 4107–4133. doi: 10.1016/j.neuron.2025.09.028, 41092898 PMC12667238

[ref57] Tracey I. Ploghaus A. Gati J. S. Clare S. Smith S. Menon R. S. . (2002). Imaging attentional modulation of pain in the periaqueductal gray in humans. J. Neurosci. 22, 2748–2752. doi: 10.1523/JNEUROSCI.22-07-02748.2002, 11923440 PMC6758341

[ref58] Tsakiris M. (2017). The multisensory basis of the self: from body to identity to others. Q. J. Exp. Psychol. 70, 597–609. doi: 10.1080/17470218.2016.1181768, 27100132 PMC5214748

[ref59] Ungerleider L. G. Kastner S. (2000). Mechanisms of visual attention in the human cortex. Annu. Rev. Neurosci. 23, 315–341. doi: 10.1146/annurev.neuro.23.1.315 10845067

[ref60] Unschuld P. U. (1985). Medicine in China: A History of Ideas. Berkeley, Los Angeles, and London: University of California Press.

[ref61] Villemure C. Bushnell C. M. (2002). Cognitive modulation of pain: how do attention and emotion influence pain processing? Pain 95, 195–199. doi: 10.1016/S0304-3959(02)00007-6, 11839418

[ref62] Ward J. H. (1963). Hierarchical grouping to optimize an objective function. J. Am. Stat. Assoc. 58, 236–244. doi: 10.1080/01621459.1963.10500845

[ref63] Wilcox R. (2012). “Introduction,” in Introduction to Robust Estimation and Hypothesis Testing, (Amsterdam: Elsevier), 1–22.

[ref64] Zunic J. Rosin P. L. (2004). A new convexity measure for polygons. IEEE Trans. Pattern Anal. Machine Intell. 26, 923–934. doi: 10.1109/TPAMI.2004.19, 18579950

