# Supplementary material for: When somatic attention creates sensation: a new class of bodily experience
Source: Front Hum Neurosci. 2026 Aug 18;20:1884618. doi: 10.3389/fnhum.2026.1884618 (PMC13529457; doi:10.3389/fnhum.2026.1884618)
Supplement: Supplementary file 1 [file Data_Sheet_1.docx]

Supplementary Material

## 1.1 Glossary

**Awareness.** A general, non-selective state of being conscious of experiences as they arise.

**Attention.** The deliberate directing of awareness to a particular aspect of experience.

**Somatic attention.** The deliberate directing of attention into the body.

**Focused somatic attention.** Somatic attention focused to a specific body region.

**Sensation.** Any consciously perceived experience.

**Physical (bodily) sensation.** A sensation experienced as located in the body, distinct from emotions, moods, or abstract thoughts.

**Referred sensation.** A physical sensation perceived at a site other than the point of stimulation or attentional focus.

**Attention-related (bodily) sensation.** A physical sensation that arises in connection with the deliberate directing of attention, independent of external stimulation.

**Attention-related referred sensation.** An attention-related sensation perceived at a site other than the point of attentional focus.

## 1.2 Standardized Verbal Instructions

To ensure consistency and reduce variability in participant experiences, we implemented a standardized set of verbal instructions across all experimental conditions. These were read aloud to the participants by the investigator.

Participants of the FSA group received the following instructions (in German) at the start of each period, when the attention anchor was placed at a new location:

*"You will float for 10 minutes with the ring, tune into yourself and notice what physical sensations you feel, if any, without interpreting them. Afterwards, I will hand you the tablet PC, and you will draw everything you felt."*

Participants of the NSA group received the following instructions just before their floating session:

*‘I am researching the health effects of floating and specific physical sensations. I would be glad if you would participate in a study on this today. Later, I would like to ask you about any physical sensations you may have experienced during today's float.*

*For that, it would be helpful if, while floating, you occasionally tune into yourself and notice any sensations you may have. You will then be asked to draw these physical sensations on a body outline using a tablet PC.’*

Finally, participants of the PSA group were asked the following after their float session: *‘*

*I am researching the health effects of floating and specific physical sensations. I would be glad if you could briefly participate in a study and answer one question for me.*

*The question is: Did you notice any physical sensations during your time in the float tank?’*

This final question was posed in identical form to participants in the NSA group immediately after their float session. In both groups, participants who answered "yes" were then asked, *‘What physical sensations did you experience during your time in the floating tank?’* before being handed the tablet PC to collect their sensation drawings.

## 1.3 Verbal Descriptors with their German versions

The German versions of the descriptors used for the sensation drawings are shown in the following list: “Tingling” (“Kribbeln”), “Numbness” (“Taubheit”), “Warm” (“Wärme”), “Dull Pain” (“Dumpfer Schmerz”), “Heaviness” (“Schwere”), “Soreness” (“Wie Muskelkater”), “Sharp Pain” (“Scharfer Schmerz”), “Pricking” (“Piksen”), “Fullness” (“Fülle”), “Throbbing” (“Pochen”), “Aching” (“Schmerz”), “Spreading” (“Sich ausbreitend”), “Pressure” (“Druck”), “Electric” (“Elektrisch”), “Distended” (“Gedehnt”), “Stinging” (“Stechen”), “Twinge” (“Zwicken”), “Pulling” (“Ziehen”), “Radiating” (“Ausstrahlend”), “Cold” (“Kälte”), and “Other Term” (“Anderer Begriff”).

## 1.4 Verbal instructions of the SymptomMapper App

When the tablet PC was first introduced to participants, they were given instructions on its functions and the body outline used to sketch sensations. The SymptomMapper app displayed the following instructions: *“Start with the sensation you perceived most clearly. Select all terms from the list that describe this sensation. Then, choose the intensity of your sensation from the color scale at the bottom of the screen. You can change this value within the drawing as often as you like. Begin your drawing by coloring the areas in the body outline where you felt the sensation. Please use both body views and pay attention to left and right!*

*For drawing, you can either outline the sensation (which will be automatically filled in) or select one of the other drawing tools by tapping on the corresponding icon. You can zoom in using two fingers to enlarge the area for more precise drawing. If you wish to create additional drawings with other terms, click „new term“ in the upper left corner. Please avoid shading, marking with X's, or using symbols.”*

## 1.5 Results of the proficiency check

Participants in the FSA group were evaluated on their proficiency with the SymptomMapper App by asking them to reproduce three standardized sensations using descriptors and localization provided on a printout. As shown in **Suppl. Fig. 1**, every participant placed the sensations on the right body view in the correct anatomical locations. Forty-eight participants correctly identified all three descriptors for their drawings, while the remaining two participants each selected two out of the three descriptors.

**Supplementary Figure 1.** Results of the drawing proficiency check. (A) Standardized shapes and descriptors that participants were asked to reproduce using the SymptomMapper app. (B) Averaged images of test drawings and descriptor choices indicating that participants were proficient in using the app.

**Supplementary Figure 2. Representative individual drawings of shape clusters.** Clustering of shape features of attention-related referred sensations in the FSA group revealed two categories: (A) elongated (linear) and (B) compact (irregular) shapes. For averaged distributions see **Fig. 5**.

**Supplementary Table S1.** Age and key outcome measures in the Focused Somatic Attention (FSA) and Natural Control (NC) groups.

**(A) Group comparison, unadjusted and age-adjusted.**

| **Measure** | **FSA (n = 50)** | **NC (n = 50)** | **Unadjusted test** | **Age-adjusted** |
| --- | --- | --- | --- | --- |
| Age, years (mean ± SD) | 25.6 ± 5.0 | 34.6 ± 8.9 | Welch t = −6.22, p = 2.4 × 10^−8^, d = −1.24 | — |
| Sensations per experience (median [IQR]) | 2.25 [1.81–2.75] | 1.00 [1.00–2.00] | U = 1995, p = 1.5 × 10^−7^, r = 0.60 | group p = 4.0 × 10^−5^; age p = 0.14 |

**(B) Within-FSA associations between age and key outcomes (Spearman rank correlation).**

| **Outcome (within FSA, n = 50)** | **Spearman ρ** | **p** |
| --- | --- | --- |
| Sensation count (mean per trial) | +0.04 | 0.77 |
| Proportion of referred sensations | +0.13 | 0.36 |
| Mean sensation intensity | +0.22 | 0.12 |

**Notes.** NC = pooled Natural Somatic Attention and Passive Somatic Awareness groups. Sensation count is the mean number of sensations per sensory experience (per-trial mean) per participant. The age-adjusted group comparison used Quade's rank-based ANCOVA (rank-transformed outcome and covariate): the FSA–NC difference in sensation count remained significant with age in the model, while age was not a significant predictor. Within the FSA group, age showed no significant association with sensation count, the proportion of referred sensations, or mean intensity, indicating that the characteristic FSA response is not driven by participant age.

All FSA participants reported sensations in every trial (responder rate = 100%); an age gradient for responder rate is therefore not estimable within the FSA group. For reference, within the NC group age was weakly and non-significantly associated with sensation count (Spearman ρ = −0.28, p = 0.05). Referred classification follows the analysis pipeline; proportion referred is the per-participant fraction of sensations classified as referred.
